# Expression analysis of the long non-coding RNA antisense to Uchl1 (AS Uchl1) during dopaminergic cells' differentiation *in vitro* and in neurochemical models of Parkinson's disease

**DOI:** 10.3389/fncel.2015.00114

**Published:** 2015-04-01

**Authors:** Claudia Carrieri, Alistair R. R. Forrest, Claudio Santoro, Francesca Persichetti, Piero Carninci, Silvia Zucchelli, Stefano Gustincich

**Affiliations:** ^1^Area of Neuroscience, International School for Advanced Studies (SISSA)Trieste, Italy; ^2^Division of Genomic Technologies, RIKEN Center for Life Science TechnologiesYokohama, Japan; ^3^Dipartimento di Scienze della Salute, Universita' del Piemonte OrientaleNovara, Italy

**Keywords:** antisense transcription, long non-coding RNA, Parkinson's disease, Nurr1, dopaminergic cells

## Abstract

Antisense (AS) transcripts are RNA molecules that are transcribed from the opposite strand to sense (S) genes forming S/AS pairs. The most prominent configuration is when a lncRNA is antisense to a protein coding gene. Increasing evidences prove that antisense transcription may control sense gene expression acting at distinct regulatory levels. However, its contribution to brain function and neurodegenerative diseases remains unclear. We have recently identified AS Uchl1 as an antisense to the mouse Ubiquitin carboxy-terminal hydrolase L1 (*Uchl1*) gene (AS Uchl1), the synthenic locus of UCHL1/PARK5. This is mutated in rare cases of early-onset familial Parkinson's Disease (PD) and loss of UCHL1 activity has been reported in many neurodegenerative diseases. Importantly, manipulation of UchL1 expression has been proposed as tool for therapeutic intervention. AS Uchl1 induces UchL1 expression by increasing its translation. It is the representative member of SINEUPs (SINEB2 sequence to UP-regulate translation), a new functional class of natural antisense lncRNAs that activate translation of their sense genes. Here we take advantage of FANTOM5 dataset to identify the transcription start sites associated to S/AS pair at Uchl1 locus. We show that AS Uchl1 expression is under the regulation of Nurr1, a major transcription factor involved in dopaminergic cells' differentiation and maintenance. Furthermore, AS Uch1 RNA levels are strongly down-regulated in neurochemical models of PD *in vitro* and *in vivo*. This work positions AS Uchl1 RNA as a component of Nurr1-dependent gene network and target of cellular stress extending our understanding on the role of antisense transcription in the brain.

## Introduction

Large genomic projects such as ENCODE (Derrien et al., 2012) and FANTOM (Forrest et al., 2014) have shown that the majority of the mammalian genome is transcribed, thus generating a previously underestimated complexity in gene regulatory networks. A vast repertory of different classes of transcripts includes non-coding RNAs and RNAs of Transposable Elements (TEs), such as LINE (long interspersed nuclear element) and SINEs (short interspersed nuclear element) (Katayama et al., 2005; Faulkner et al., 2009; Kapranov et al., 2010; Fort et al., 2014). Long non-coding RNA (lncRNA) genes seem to represent the majority of cellular transcriptional output. The FANTOM project has cataloged more than 30,000 putative lncRNA transcripts by full length cDNA cloning (Katayama et al., 2005) while NONCODEv4 currently contain 46,475 lncRNA genes (Xie et al., 2014; Quek et al., 2015).

Antisense (AS) transcripts are RNA molecules that are transcribed from the opposite strand to sense (S) genes forming S/AS pairs. These are estimated to include the large majority of protein encoding genes and about one third of lncRNAs (Chen et al., 2004; Katayama et al., 2005; Engstrom et al., 2006; Derrien et al., 2012). The most prominent class of S/AS pair is when a protein-encoding gene presents a lncRNA on the opposite strand. In a growing number of cases, AS lncRNAs have been proved to be required for proper regulation of sense genes, carrying genetic information acting at distinct regulatory levels (Yu et al., 2008; Spigoni et al., 2010; Tripathi et al., 2010). Understanding their mode of action may be also relevant for gene expression manipulation *in vivo* since lncRNAs may become in the near future a new class of RNA-based drugs for therapeutic intervention.

The contribution of AS lncRNAs to neurodegenerative diseases is still unclear although some significant examples in Alzheimer's disease (AD) (Faghihi et al., 2008, 2010), spinocerebellar ataxia type 7 (Sopher et al., 2011) and Huntington's disease (Chung et al., 2011) may suggest they play a prominent role in neuronal homeostasis and dysfunction.

Parkinson's disease (PD) is a slowly progressive degenerative disorder of the central nervous system (CNS) that is classically defined in terms of motor symptoms. The neuropathological hallmark in *post-mortem* brains is the selective degeneration of specific subsets of mesencephalic dopaminergic (DA) cells and the formation of cytoplasmic aggregates called Lewy bodies. The current model of toxicity of DA neurons includes mitochondrial dysfunction, oxidative stress and alterations in protein turnover. This stems from the observation on *post-mortem* PD brains as well as from the identification of genes associated to rare forms of early-onset familial PD. Some of these features are recapitulated in a neurochemical model of the disease that takes advantage of the selective accumulation of toxic MPP^+^ species in DA neurons.

So far, relevant examples for lncRNAs antisense to genes involved in PD have been restricted to a transcript associated to PINK1/PARK6 locus (Scheele et al., 2007).

Recently, we have identified a lncRNA that is antisense to the mouse Ubiquitin carboxy-terminal hydrolase L1 (*Uchl1*) gene (AS Uchl1), the synthenic locus of UCHL1/PARK5 (Carrieri et al., 2012).

Uchl1 encodes for one of the most abundant proteins in the brain. It acts as deubiquinating enzyme, ubiquitin ligase or monoubiquitin stabilizer, thus regulating ubiquitin turnover (Liu et al., 2002). Dysfunction in UCHL1 has been reported in many neurodegenerative diseases. A missense mutation in UCHL1/PARK5 has been associated to rare cases of early-onset familial PD. Inactivating oxidative modifications of UchL1 protein have been reported in PD *post-mortem* brains where it correlates with the formation of protein aggregates (Choi et al., 2004; Barrachina et al., 2006; Gong et al., 2006). In this context several evidences position UchL1 as a major regulator of α-synuclein degradation and toxicity (Liu et al., 2009). Lack of hydrolase activity has also been found in recessive cases of a childhood-onset progressive neurodegeneration (Bilguvar et al., 2013). An in-frame deletion in the Uchl1 gene, as observed in the *gracile axonal dystrophy* mice, leads to axonal dystrophy and premature death (Saigoh et al., 1999). Reduced UCHL1 protein levels were also found in sporadic AD brains. Recently, UCHL1 overexpression has been shown to accelerate lysosomal degradation of APP, inhibit plaque formation and improve memory deficits in AD transgenic model mice (Gong et al., 2006). These data proves UchL1 activity is required for proper brain function. Furthermore, they suggest that increasing UCHL1 expression *in vivo* may be a safe and effective disease-modifying strategy to treat neurodegenerative diseases. It is therefore important dissecting all the molecular events involved in Uchl1 gene regulation.

AS Uchl1 is a 5′ head to head, 1.2 kb long transcript that initiates within the second intron of Uchl1 and overlaps the first 73 nts of the sense mRNA including the AUG codon. The non-overlapping part of the transcript also contains an embedded repetitive sequence SINEB2 of the B3 subclass in the inverted orientation. AS Uchl1 is expressed in mouse mesencephalic DA neurons, the site of degeneration in PD. In physiological conditions AS Uchl1 RNA is nuclear-enriched. Upon rapamycin, it shuttles from the nucleus to the cytoplasm and specifically targets Uchl1 mRNA to heavy polysomes for translation (Carrieri et al., 2012). AS Uchl1 is the representative member of SINEUPs (SINEB2 sequence to UP-regulate translation), a new functional class of natural antisense lncRNAs that activate translation of their sense genes (Zucchelli et al., submitted).

Cap Analysis of Gene Expression (CAGE) is a technology based on the generation of short sequence tags from the 5′ end of full-length cDNAs followed by high-throughput sequencing. When mapped to a reference genome, CAGE tags survey transcription start site (TSS) activity of specific promoters and measure expression levels on a massive scale (Gustincich et al., 2003; Shiraki et al., 2003; Carninci et al., 2006). The FANTOM5 (Functional Annotation of Mammals 5) project has developed a simplified CAGE protocol adapted to single-molecule HeliScope sequencer (hCAGE) (Kanamori-Katayama et al., 2011) to decrease PCR biases and improve depth of sequencing. hCAGE technology was applied to a wide range of human and mouse tissues providing an unprecedented dataset for promoter usage analysis (Forrest et al., 2014).

Here we take advantage of FANTOM5 dataset to map TSSs and analyze the expression of the S/AS pair at the Uchl1 mouse locus. This led to the identification in the AS Uchl1 promoter region of a binding site for Nurr1, a transcription factor required for DA cells differentiation. Chromatin immuno-precipitation and quantitative RT-PCR proved that AS Uchl1 expression is under the control of Nurr1 activity. Finally, we show that transcription of S/AS Uchl1 RNA is regulated in neurochemical models of PD *in vitro* and *in vivo*.

## Materials and methods

### Cell lines

Murine dopaminergic MN9D cells with doxyciclin- inducible induction of Nurr1 transcription factor (MN9D Nurr1 Tet-ON stable cells, or iMN9D cells) were maintained in culture as previously described (Hermanson et al., 2003). Nurr1 expression was obtained by culture with 2.5 μg/ml doxycycline for 12 h or longer, as required. For *in vitro* neurochemical model of PD, iMN9D cells were treated with 100 μM MPP^+^ for 16 h.

### ChIP assay

Chromatin immunoprecipitation (ChIP) was performed with magnetic beads (Dynabeads, Invitrogen) following the protocol as described (Schmidt et al., 2009). For each ChIP, one confluent 100 mm plate of iMN9D cell was treated with doxycicline 2.5 μg/ml. Nurr1 expression upon doxycicline treatment was followed by western blot. 1 μg of ChIP-grade anti-Nurr1 antibody was used (sc-990 X). Rabbit IgG were used as negative control (Cell signaling #2729).

qPCR was performed with primers for DNA binding regions of indicated targets and distal primers were designed for an unrelated region 6000 bps upstream the AS Uchl TSS:

VMAT NBRE F: 5′-ATTGTGCTAACATTTATTCCAGAG-3′

VMAT NBRE R: 5′-AGGGCTTCCTACGTGACC-3′

OCN NBRE F: 5′-CCACAACACGCATCCTTT-3′

OCN NBRE R: GGACTTGTCTGTTCTGCA-3′

AS Uchl1 NBRE F: 5′ CTTCCCATACAGCTTAGTTCC-3′

AS Uchl1 NBRE R 5′-TTGCGTCTCTGCCAGATG-3′

Distal F 5′-TCATCCAGCCACAAGGTCAGAG-3′

Distal R 5′- CCAGCAGGCACACTGTTGAAC-3′

Enrichment of chromatin binding was calculated relative to total input, as described previously (Guccione et al., 2006).

### RNA isolation, reverse transcription and quantitative RT-PCR (qRT-PCR)

Total RNA was extracted from iMN9D cells or dissected mouse ventral midbrain using TRIZOL reagent (Invitrogen) and following manufacturer's instructions. RNA was treated with DNAse I (Ambion) before use. Single strand cDNA was prepared from 1 μg of purified RNA using the iSCRIPT™ cDNA Synthesis Kit (Bio-Rad) according to manufacturer's instructions. qRT-PCR reaction was performed using SYBR-Green PCR Master Mix (Applied Biosystem) and an iCycler IQ Real time PCR System (Bio-Rad). Oligonucleotide sequences of primers used in this study were previously described (Carrieri et al., 2012). qRT-PCR for Nur77 was performed with the following primers:

Nur77-FWD CCTCATCACTGATCGACACG

Nur77-REV CCTCCAACTTGAGGCAAAAG

### MPTP treatment

Mice used in this study were treated according to the NIH guidelines for Care and Use of Laboratory Animals. MPTP use and safety precautions were as described previously (Jackson-Lewis and Przedborski, 2007). All animal experiments were performed in accordance with European guidelines for animal care and following SISSA Ethical Committee permissions. Mice were housed and bred in SISSA non-SPF animal facility, with 12 h dark/light cycles and controlled temperature and humidity. Mice had *ad libitum* access to food and water.

Eight-week-old, male, TH-GFP mice (Sawamoto et al., 2001) were subjected to a sub-acute MPTP regimen (Jackson-Lewis and Przedborski, 2007). Animals received one intra-peritoneal injection of MPTP-HCl (free base suspended in saline; Sigma-Aldrich) at 20 mg kg^−1^ dose every 2 h for a total of four doses over an 8 h period (Tatton and Kish, 1997; Miller et al., 2004; Gibrat et al., 2009). Injection of saline solution was used in control mice. Animals were sacrificed 2 days or 7 days after last injection, as these time-points were previously associated to variations in Uchl1 and Nurr1 expression (Miller et al., 2004; Gibrat et al., 2009). 300 DA neurons were purified by laser capture microdissection as described previously (Biagioli et al., 2009; Carrieri et al., 2012) and used for RNA extraction and qRT-PCR.

### Bioinformatic analysis

Analysis of FANTOM5 collection of mouse libraries was performed using the Zenbu browser genomic tool (Severin et al., 2014) and publicly available FANTOM5 datasets (http://fantom.gsc.riken.jp/5/) (Forrest et al., 2014). A specific script was designed to extract expression values from graphical tables in Zenbu Genome Browser and convert into Excel spreadsheet for further analysis (Paolo Vatta, unpublished). Expression values for S/AS Uchl1 were calculated for a window of about ±800 base pairs around main TSS. Selection of brain-specific libraries was done by manual annotation.

For co-expression analysis, average expression values were calculated for S/AS transcripts in libraries that express both and divergence from average was considered.

For ChIP experiments, identification of NGFI-B binding elements was performed with the Genomatrix program (http://genomatrix.de) and the TRANSFAC database (Knuppel et al., 1994). The mouse *AS Uchl1* genome region from kb −3000 to +1000 was the reference sequence. Trascriptional binding factor motifs were chosen on the basis of core similarity (score 1.0) and matrix similarity (above 0.80).

## Results

### Evidence of as Uchl1 transcription in mouse FANTOM5 collection of cell lines, primary cells and tissues

We first interrogated FANTOM5 expression data for almost 400 mouse samples, covering cell lines, primary cells and tissues. These dataset are build from hCAGE libraries and are based on sequencing cDNA copies of the 5′ ends of mRNAs, of which the integrity is inferred by the presence of their cap. These sequences—referred to as *tags*—are sufficiently long to be aligned in most cases at a single location in the genome. The first position of this alignment identifies a base pair where transcription is initiated defining a TSS. The number of times a given tag is represented in a library gives an estimate of the expression level of the corresponding transcript.

**Figure 1:**
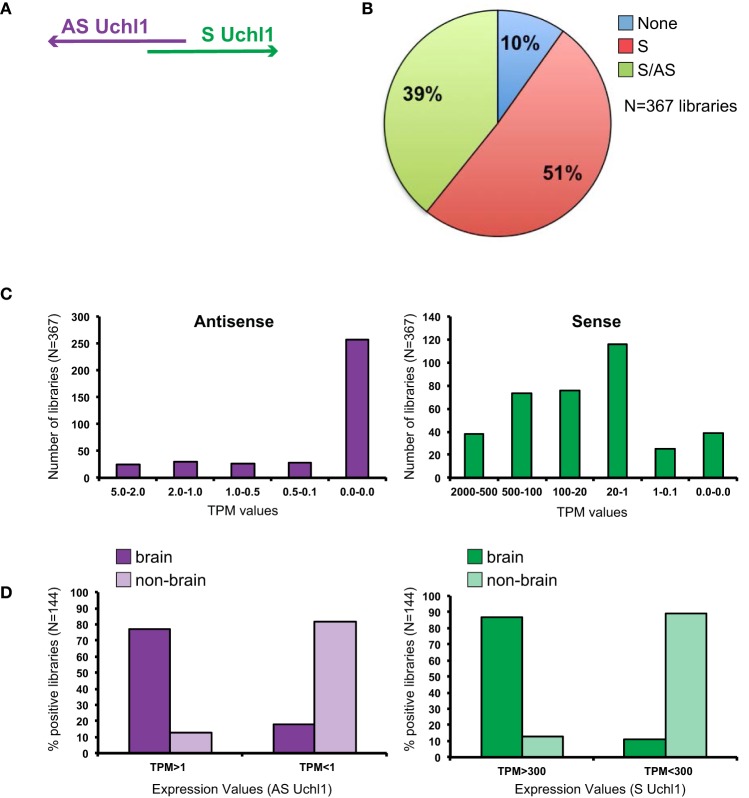
**General features of S/AS Uchl1 expression in mouse FANTOM5 datasets**. **(A)** Schematic view of S/AS Uchl1 pair with 5′ head-to-head divergent anatomy. **(B)** FANTOM5 collection of 367 libraries from mouse cell lines, primary cells and tissues were interrogated for expression of *Uchl1* gene in sense and antisense orientation. Pie-chart representation of S/AS expression in FANTOM5 dataset. **(C)** Distribution of S/AS levels in mouse libraries. Transcript levels are expressed as tag per millions (TPM). **(D)** Higher levels of S and AS Uchl1 are enriched in the brain. Data were obtained from *N* = 144 FANTOM5 libraries that express both transcripts. Low and high levels of S and AS Uchl1 were defined based on average expression values for each transcript.

Since antisense lncRNAs are typically expressed at much lower level than overlapping protein-coding transcripts (Derrien et al., 2012; Forrest et al., 2014) (Zucchelli et al., FANTOM5 satellite, submitted), we used FANTOM5 mouse datasets in which no expression cutoff was applied to detect values as low as 1 count per library. Tags were positively scored if mapping to a region of about 800 bp around the main TSS of Uchl1 (Figures 1A, **3**). Expression was measured as Tag Per Million (TPM).

While sense mRNA is expressed in almost all analyzed samples (90% of libraries), only 40% of them score positive for antisense transcription proving AS Uchl1 expression is relatively restricted (Figure 1B). Sense mRNA is present at higher levels as compared to its antisense (Figure 1C). Both transcripts are highly expressed in the brain (Figure 1D). In particular AS Uchl1 RNA is present in the cortex, striatum and hippocampus (TPM 3.2-2.2) although its highest level is measured in testis (TPM 5.38). Within FANTOM5 dataset, about 50 libraries were prepared from blood-derived primary cells (B cells, T cells, macrophages, lymphoid and myeloid progenitor cells, megakaryocyte precursor cells) and tissues related to immune system (spleen and thymus). Very low expression is detected for AS Uchl1 (0.2 TPM) in only one/two of the replicas from naïve CD4 T cells, c-Kit+ stem cells and common myeloid progenitors (data not shown).

To assess whether transcription in opposite orientation is co-regulated, we analyzed FANTOM5 libraries containing tags for both RNAs (*N* = 144 libraries). Since Spearman Correlation analysis could not be applied, we calculated average TPM levels for each transcript and set this value to 1. We then normalized expression values in all libraries to the reference and monitored variation from the average. We found that the majority of libraries (*N* = 122) displays concordant variation of S/AS transcription from the average, with both positive and negative co-regulation. When expression of S/AS pair is not co-regulated (*N* = 22), variation from average values goes in both directions and is almost equally present in non-brain (*N* = 9) and brain (*N* = 13) libraries.

**Figure 2:**
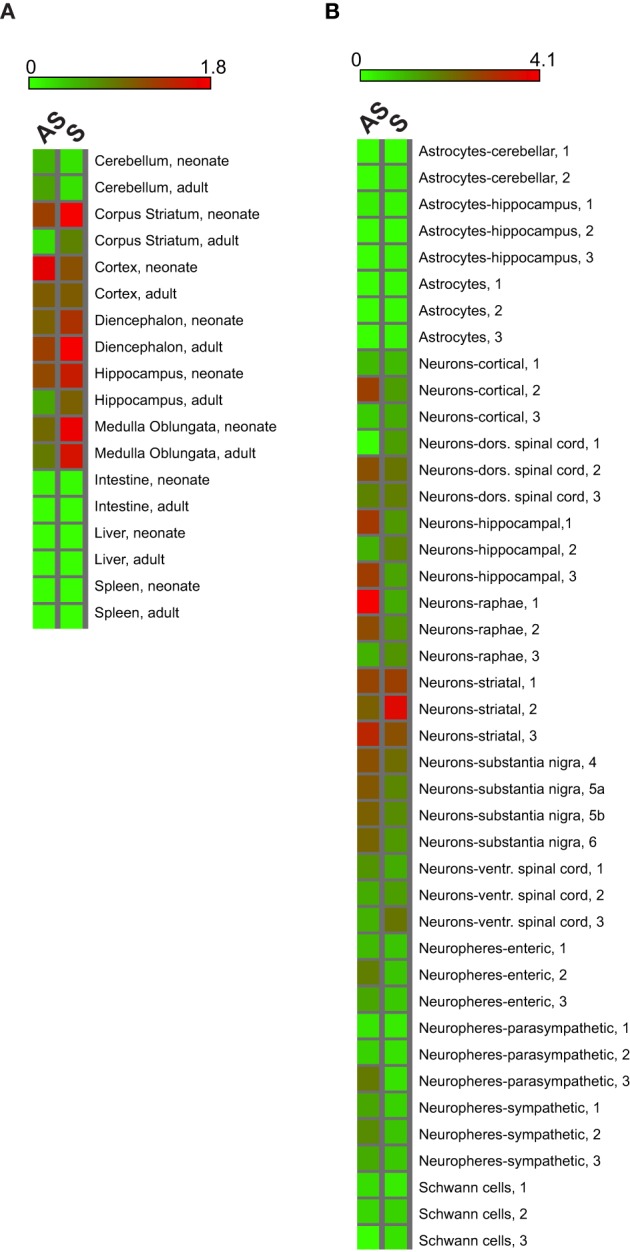
**S/AS Uchl1 are regulated during development and enriched in neurons**. **(A)** Heat map graphical representation of S/AS expression in neonatal and adult tissues from brain and other organs. Expression values of S/AS transcripts in cortex were set to 1. **(B)** Heat map of S/AS Uchl1 in brain-derived primary cells and in neurospheres. Values in cortical neurons were set to 1.

Since FANTOM5 collection comprises a set of brain regions for which neonatal and adult tissues are available, we monitored S/AS transcription in brain development. As previously found, sense and antisense levels are positively co-regulated with values in neonatal tissues often higher than in adult samples of the same area (Figure 2A). High levels of AS Uchl1 RNA are detected in neonatal corpus striatum, cortex, hippocampus and medulla oblungata.

As each brain region is composed of several cellular types of neuronal and non-neuronal origin, we monitored S/AS expression in primary brain cells as well as in neurospheres. Again, we found that the expression of S and AS Uchl1 RNAs is co-regulated. Both transcripts are highly expressed in neurons as compared to non-neuronal cells, with those from the Striatum, the *Substantia Nigra* and Raphe being the highest (Figure 2B). Interestingly, values in neurospheres prepared from cells isolated from different compartments show reduced quantities as compared to primary neurons. Non-neuronal cells have very low (Schwann cells) to undetectable (astrocytes) levels of Uchl1 S/AS pair.

Therefore, expression of S and AS Uchl1 is co-regulated in the developing and adult brain.

### Analysis of TSS usage in mouse Uchl1 gene locus

**Figure 3:**
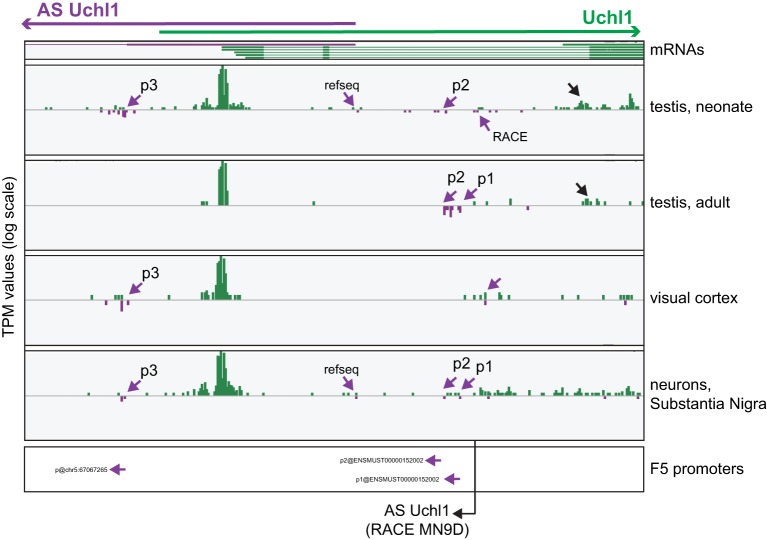
**Analysis of TSS usage in mouse S/AS Uchl1**. Zenbu Genome Browser view of mouse Uchl1 locus. Sense transcripts are in green, antisense in purple. hCAGE data are presented for those libraries (testis, cortex and *Substantia Nigra* neurons), where antisense expression is highest. Purple arrows highlight antisense TSSs identified by FANTOM5 data analysis (p1, p2, and p3, 5′ to 3′ orientation relative to AS Uchl1) and by RACE experiments in MN9D cells. TSS supporting the expression of RefSeq AS Uchl1 transcript in testis and *Substantia Nigra* is also indicated. Promoters identified with FANTOM5 decomposition-based peak identification method are shown at the bottom (original promoter nomenclature is given). Black arrow indicated an alternative transcript variant of sense Uchl1. The 5′ end of AS Uchl1 cDNA cloned from mouse dopaminergic MN9D cell line is shown (Carrieri et al., 2012).

To monitor promoter usage in mouse Uchl1 locus, we visualized single TSSs using the FANTOM5 Zenbu Genome Browser, a publicly available web resource tool (http://fantom.gsc.riken.jp/zenbu/) (Severin et al., 2014). To identify promoters across the genome the FANTOM5 consortium has developed a method based on tags proximity and signal decomposition (Forrest et al., 2014). To enrich for TSSs, 3 tags/library thresholds were applied and promoter subsets were defined and classified according to their robustness. For our analysis, we interrogated all hCAGE libraries pooled together and then we focused our attention on those with stronger evidences of TSS usages, as testis, cortex and primary neurons of the *Substantia Nigra*. As shown in Figure 3, three different TSSs can be identified using decomposition-based identification method and referred to as p1, p2 and p3, from 5′ to 3′ according to the sense of transcription. These drive expression of alternative variants of AS Uchl1 RNA. TSS usage is under tissue- and cell-specific regulation, as documented by different TPM values in the selected libraries. Interestingly, expression of AS Uchl1 reference sequence is validated by TSSs in testis and *Substantia Nigra* neurons, but this promoter does not pass the bioinformatics cutoffs. p2 and p1 are the most used promoters, giving rise to transcript variants with a longer 5′ end. It is of note that 5′ end of AS Uchl1, as identified by RACE in murine dopaminergic MN9D cell line (Carrieri et al., 2012), is positioned upstream to p1 (Figure 3). Finally, an additional site of transcription initiation can be observed at the 3′ end of AS Uchl1 (p3), thus identifying a yet not-annotated variant with a shorter first exon. p3 seems to be under control of a bidirectional promoter.

The protein-coding Uchl1 mRNA starts almost exclusively from an internal portion of the 5′ UTR of the annotated transcript. It is shorter and independently validated by mm9 mRNAs (Figure 3). Interestingly, an alternative promoter for Uchl1 is also located around exon 3 and drives expression in testis (Figure 3, black arrow). This TSS identifies an annotated but yet uncharacterized transcript (AK170728), which potentially encodes for a shorter Uchl1 protein, lacking a canonical initiation methionine. Whether such protein is indeed functional remains to be established.

### Nurr1 activity regulates as Uchl1 expression

**Figure 4:**
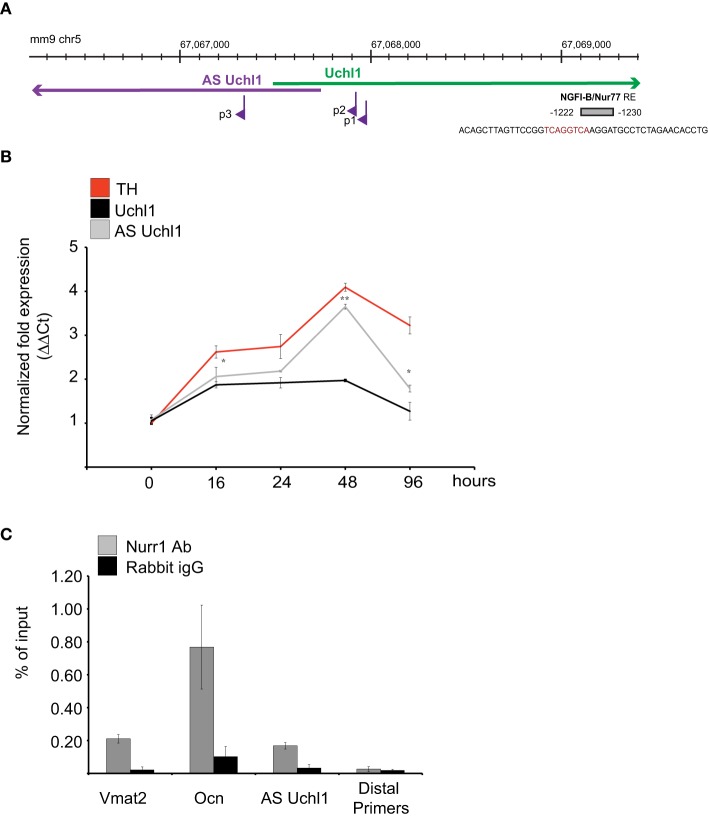
**AS Uchl1 is primary target of Nurr1 transcription factor**. **(A)** Scheme of Nurr1 TFBS upstream AS Uchl1. Genomic coordinates (top), S/AS Uchl1 transcript anatomy (middle), and AS Uchl1 promoters (bottom) are shown. NGFI-B/Nur77 response element (NBRE) is indicated in gray and positioned to its genomic coordinates and relative to AS Uchl1 TSSs. **(B)** AS Uchl1 expression is up-regulated during differentiation. iMN9D cells were treated with doxocyclin at the indicated times. AS and S Uchl1 expression were monitored by qRT-PCR. Expression of TH was used as positive control for dopaminergic differentiation. ^*^p < 0.05; ^**^p < 0.01. **(C)** Immunoprecipitation of Nurr1-bound chromatin was performed from murine dopaminergic iMN9D cells treated with doxocyclin for 12 h to induce Nurr1 expression. Rabbit IgGs and distal primers targeting an unrelated region were used as controls. Chromatin immunoprecipitation of VMAT2 and OCN promoter regions were included as positive controls of Nurr1 binding.

The genomic region −1500/+1000 around the annotated AS Uchl1 TSS was then scanned for the presence of Transcription Factor Binding Sites (TFBSs). A NGFI-B element was identified in position −1230/−1222 to the AS Uchl1 TSS as defined by RACE (Figure 4A). This TFBS is the target of the Nurr subfamily of nuclear receptors including Nurr1, a key dopaminergic transcription factor required for late-dopaminergic differentiation and crucial for the expression of several dopaminergic-specific genes like VMAT2, AADC, DAT, and TH (Castro et al., 2001).

To study the role of this transcription factor in AS Uchl1 RNA transcription, we took advantage of mouse dopaminergic MN9D cell line overexpressing Nurr1 under a doxycicline inducible promoter (iMN9D cells) (Hermanson et al., 2003). In steady-state conditions, levels of Nurr1 protein are almost undetectable and AS Uchl1 is expressed at low level. Upon drug treatment, Nurr1 expression increases and Nurr1 target genes are induced (Hermanson et al., 2003). As a consequence, differentiation of DA neurons occurs (Figure 4B).

We then carried out chromatin immunoprecipitation (ChIP) experiments. iMN9D cells were treated with doxocycline for 12 h to achieve high levels of Nurr1 protein. Chromatin-protein complexes were then immunoprecipitated with anti-Nurr1 or control (IgG) antibodies and bound genomic DNA was quantified by qPCR using primers for Nurr1 response elements in AS Uchl1, Osteocalcin and Vescicular Monoamine Transporter 2 gene promoters. PCR reactions generated only the expected specific amplicon, as detected by gel electrophoresis and melting curve analysis (data not shown). As shown in Figure 4C, Nurr1 binding was significantly enriched relative to IgG control in AS Uchl1 promoter region.

Nurr1 induction led to a rapid up-regulation of AS Uchl1 RNA levels starting from 12 h, with kinetics comparable to the one observed for VMAT2, a well-known primary target of Nurr1. Within 48 h, both AS Uchl1 and VMAT2 mRNAs reached their peak of induction, while decreasing at later time points. Under these conditions, Nur77, another member of nuclear receptors family targeting NGFI-B motif, is undetectable (Supplementary Figure 1).

Interestingly, we found that transcription of sense protein-coding Uchl1 mRNA was poorly linked to Nurr1 activity, as the kinetics and strength of Uchl1 mRNA up-regulation was different from that of VMAT2 and AS Uchl1 (Figure 4B).

### Expression of mouse as Uchl1 is down-regulated in neurochemical models of PD *in vitro* and *in vivo*

**Figure 5:**
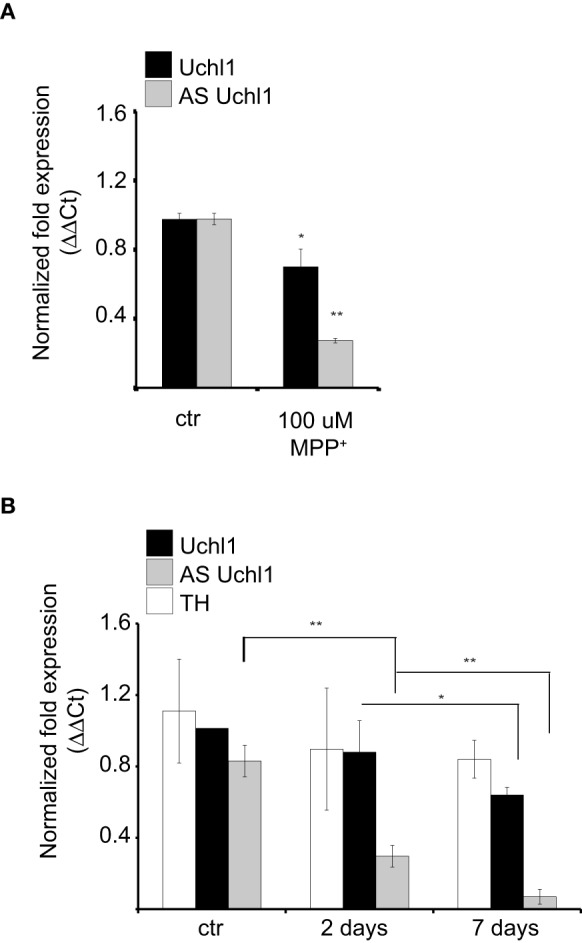
**S/AS Uchl1 pair is co-downregulated in PD neurochemical models *in vitro* and *in vivo*. (A)** qRT-PCR of S and AS Uchl1 transcript levels in iMN9D cells treated with 100 μM MPP^+^ for 16 h. ^*^p < 0.05; ^**^p < 0.01. **(B)** TH-GFP mice were treated with MPTP or saline control (ctrl). Animals were sacrificed at the indicated times. RNA was extracted from LCM-purified DA neurons and used for qRT-PCR. Data indicate mean ± s.d., *n* = 8. ^*^p < 0.05; ^**^p < 0.01.

We then investigated the behavior of Uchl1 S/AS pair in neurochemical models of PD. First, we analyzed the effects of intoxication on S/AS Uchl1 levels *in vitro*. When iMN9D cells were exposed to 100 μM 1-methyl-4-phenylpyridinium (MPP^+^) for 16 h, we observed that expression of both transcripts was altered and a statistically significant co-reduction could be measured. While AS Uchl1 RNA was reduced to only 25% of its physiological level, the impact on Uchl1 mRNA was less pronounced, with only 30% reduction (Figure 5A).

To test whether S/AS transcription is regulated during DA neuron intoxication *in vivo*, we injected mice with 1-methyl-4-phenyl-1, 2, 3, 6-tetrahydropyridine (MPTP), following a sub-acute protocol (Jackson-Lewis and Przedborski, 2007). To uncouple transcriptional control of S/AS expression from down-regulation due to cell loss, we resolved to laser capture microdissection of fluorescently labeled DA neurons from TH-GFP transgenic mice to purify equal numbers of cells from untreated and treated animals. Under these conditions, surviving DA neurons showed an almost complete loss of AS Uchl1 transcript but a modest down-regulation of Uchl1 mRNA (Figure 5B) recapitulating what we had observed *in vitro* in iMN9D cells treated with MPP^+^.

Altogether, these data show that AS Uchl1 is a component of the Nurr1-dependent dopaminergic gene network and is down-regulated in neurotoxicity.

## Discussion

One of the key features of genomes' organization is that the large majority of genes share their genomic region with another gene on the opposite filament forming S/AS pair (Carninci et al., 2005; Derrien et al., 2012). Massive antisense transcription seems to be a common feature of cells ranging from bacteria to human (Katayama et al., 2005; Van Dijk et al., 2011). Despite some provocative examples we poorly understand how each locus decides between repressive and activating responses to antisense transcription suggesting we are still missing the basic principles of regulatory choices and molecular switches. This is particularly relevant when we consider that lncRNAs and antisense transcription may be exploited in RNA therapeutics.

The S/AS pair at the Uchl1 locus is organized according to the configuration where a protein-encoding gene presents a lncRNA on the opposite strand, overlapping “head-to-head” at their 5′ ends. AS Uchl1 is the representative member of SINEUPs, a new functional class of natural and synthetic antisense lncRNAs that activate translation. Their activity depends on the combination of two RNA elements: the overlapping region is indicated as the Binding Doman (BD) while the embedded inverted SINEB2 element is the Effector Domain (ED). The ED is required for the increase of translation of the protein-coding sense mRNA and the BD is targeting the activity to the sense transcript.

UchL1 is one of the most abundant proteins in the brain where it is expressed in selected neuronal cell types. We have previously shown that in iMND9 cells, the very same cell line used in this study, its mRNA is associated to light polysomes for basal translation. In these conditions AS Uchl1 is mainly restricted to the nucleus where it exerts an unknown function. S/AS transcripts are also co-expressed in mesencephalic DA cells *in vivo* where they retain their differential subcellular localization. Upon inhibition of mTOR activity by rapamycin, AS Uchl1 shuttles to the citoplasm where it facilitates Uchl1 mRNA loading to heavy polisomes for a more efficient translation and increase in cellular UchL1 protein levels. Both rapamycin treatment and Uchl1 over-expression are considered neuroprotective suggesting that antisense-mediated translation may be part of a pro-survival cellular response to stress.

A first step toward a better understanding of antisense transcription consists in studying how its expression is regulated. Increasing information is accumulating over the role of DNA methylation and selected TFs such as Sox2, Oct4, and Nanog on the control of long intergenic non-coding RNAs (lincRNAs) (Guttman et al., 2009) whereas very little is known about the transcriptional regulation of the lncRNAs component of S/AS pairs. Recent genome-wide studies on human cell lines evidence epigenetic regulation of AS lncRNAs expression, with accumulation of histone marks and RNA polymerase II occupancy (Conley and Jordan, 2012). Interestingly, TSSs in antisense orientation frequently associate with transposable elements, suggesting that repetitive sequences might contribute to regulation of AS transcription (Conley et al., 2008; Kapusta et al., 2013). Few gene-specific examples exist in which transcriptional control of a natural AS transcript has been investigated. This is the case, for example, of S/AS Msx1 in which the promoter region of AS RNA contains binding sites for Msx1 transcription factor, thus generating an auto-regulatory loop of S/AS regulation (Petit et al., 2009; Babajko et al., 2011). In prostate cancer cell line, the expression of CTBP1-AS is regulated by binding of androgen receptor at the 5′ of its TSS. Upon androgen-dependent activation of CTBP1-AS, the overlapping protein-coding gene (CTBP1) is repressed (Takayama et al., 2013).

Here we find that almost all the FANTOM5 libraries present evidences of Uchl1 transcription while AS Uchl1 is expressed only in 40% of them. This data raise the question about how UchL1 protein synthesis is regulated in physiological conditions and upon stress in the absence of antisense transcription. It will be interesting to assess whether Uchl1 mRNA is normally associated to heavy polisomes and whether mTOR inhibition or stress are able to increase UchL1 protein levels according to an antisense–independent mechanism. This is particularly relevant considering that UchL1 is target of oxidative stress inactivation and is down-regulated in *post-mortem* brains of neurodegenerative diseases. In summary these data may raise the interesting possibility of differences in the anti-stress control and function of Uchl1 expression dependent on the presence of AS Uchl1 RNA.

We did not find any example of AS Uchl1 expression in the absence of Uchl1. Therefore, it is not surprising that when both S/AS pair transcripts are present they show a concordant pattern of expression. The highest levels of AS Uchl1 have been found in *Substantia Nigra*, striatum and diencephalon. This analysis confirms AS Uchl1 is present in selected regions of the brain as shown in Carrieri et al. (2012).

Interestingly, neonatal neurons express larger quantity of AS Uchl1 RNA than adult tissues. This is in line with recent genome-wide and gene-specific studies that indicate spatiotemporal regulation of AS lncRNAs expression during cerebral corticogenesis (Ling et al., 2011; Lipovich et al., 2013) and aging (Pardo et al., 2013) (Francescatto et al., FANTOM5 satellite, submitted).

Even when AS Uchl1 is highly expressed, its levels are lower than Uchl1 mRNA. However, we know that AS Uchl1 is able to increase the amount of UchL1 protein through its activity on Uchl1 mRNA. The quantity of UchL1 protein *in vivo* is the sum of several molecular events ranging from transcription to mRNA localization, rate of protein synthesis and degradation. It is unclear what is the life cycle of a single Uchl1 mRNA from transcription to its association to ribosomes or stress granules and P bodies. Therefore, it remains crucial to determine which fraction of total cellular Uchl1 mRNA is associated to light polisomes in physiological conditions and how much is available for RNA-RNA interaction and association to heavy polisomes in an antisense-dependent fashion under stress.

By decomposition-based identification method three different promoters can be defined while evidences for additional TSSs are shown. While p1 and p2 extend the overlapping region at genomic level respect to the annotated sequence of reference, a previously reported cDNA clone obtained with RACE starts 5′ to p1. p3 identifies a non-overlapping transcript probably involved in a bidirectional promoter. Interestingly, a potential internal alternative TSS for Uchl1 is evidenced in testis and *Substantia Nigra* suggesting the possibility of a N-terminal truncated protein.

At 1230 nt from the TSS of AS Uchl1 identified with RACE, a DNA binding site for Nurr1 has been bioinformatically discovered and experimentally validated. Nurr1 is a member of the nuclear receptor superfamily, whose expression is crucial for DA neurons differentiation and maintenance in the adult (Zetterstrom et al., 1997; Saucedo-Cardenas et al., 1998; Kadkhodaei et al., 2009, 2013). Importantly, reduced quantities of Nurr1 in adult mice increase DA cells vulnerability to neurotoxic insults (Le et al., 1999). In humans, Nurr1 polymorphisms have been associated to sporadic PD (Xu et al., 2002; Grimes et al., 2006) and reduced Nurr1 levels have been measured in peripheral blood and brain of PD patients (Chu et al., 2002; Liu et al., 2012). In summary, Nurr1 controls the major transcriptional axis for differentiation of DA neurons and maintenance of their integrity.

This work strongly suggests that AS Uchl1 is a component of Nurr1-dependent transcriptional network. This functional interaction adds a new layer to the complexity of circuitry that may contribute to DA cells' homeostasis.

We then prove that neurochemical intoxication of DA cells down-regulates S/AS expression *in vitro* and *in vivo* with a kinetics that impact more strongly AS Uchl1 than its overlapping sense mRNA. This is different from the consequences of pharmacological inhibition of mTOR where no effects on transcription were observed (Carrieri et al., 2012). Further studies are needed to understand whether survival or apoptotic outocomes of different treatments reside, at least in part, on the ability to regulate antisense RNAs at transcriptional or post-transcriptional levels.

In summary, antisense transcription imposes an additional level of regulatory networks to control the activity of protein-coding genes. Determining the repertory of transcription factors controlling the expression of AS RNAs under physiological and pathological conditions will further contribute to our understanding on the biological functions of AS lncRNAs in health and disease. Finally, since increasing evidences suggest Uchl1 over-expression could be beneficial in neurodegenerative diseases, the use of AS Uchl1 as RNA-based drug may represent a new therapeutic strategy.

### Conflict of interest statement

SG, PC, CC, AF, and SZ are named inventors in patent issued in the US Patent and Trademark Office on SINEUPs and licensed to TransSINE Technologies. SG and PC are co-founders of PARKscreen, an Italian SME aimed to use and develop therapeutic SINEUPs.

## Supplementary material

Supplementary Figure 1 **Nur77 expression in iMN9D cells**. iMN9D cells were treated with doxycyclin for 48 h. Expression of Nur77 was monitored by qRT-PCR with specific primers. Expression of VMAT2 mRNA was used as positive control for Nurr1-mediated activation. Data were analyzed using the 2^−Δ^Ct method. Data indicate mean ± SD. Nurr1 expression was verified by western blotting.

## Abbreviations

Uchl1: Ubiquitin Carboxy-terminal Hydrolase L1
S: sense
AS: antisense
hCAGE: Heliscope Cap Analysis of Gene Expression
TSS: transcription start site
lncRNA: long non-coding RNA
PD: Parkinson's disease
DA: dopaminergic
TH: tyrosine hydroxylase
Vmat2: vescicular monoamine transporter 2
MPP^+^: 1-methyl-4-phenylpyridinium
MPTP: 1-methyl-4-phenyl-1, 2, 3, 6-tetrahydropyridine.
