# Supplementary figures and images for: Expression analysis of the long non-coding RNA antisense to Uchl1 (AS Uchl1) during dopaminergic cells' differentiation in vitro and in neurochemical models of Parkinson's disease
Source: Front Cell Neurosci. 2015 Apr 1;9:114. doi: 10.3389/fncel.2015.00114 (PMC4381646; doi:10.3389/fncel.2015.00114)

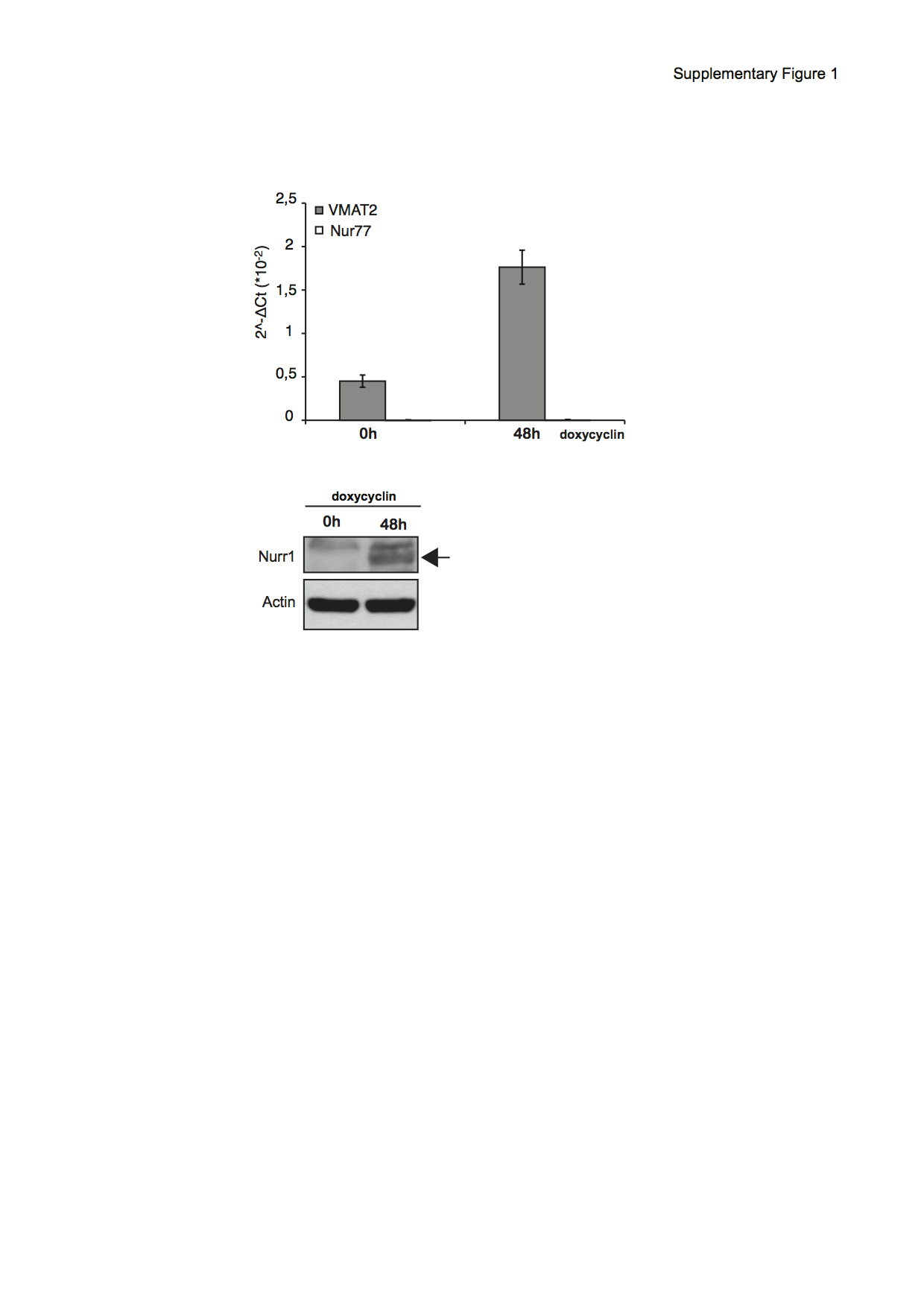

Supplement: Supplementary Figure 1 — Nur77 expression in iMN9D cells. iMN9D cells were treated with doxycyclin for 48 h. Expression of Nur77 was monitored by qRT-PCR with specific primers. Expression of VMAT2 mRNA was used as positive control for Nurr1-mediated activation. Data were analyzed using the 2−ΔCt method. Data indicate mean ± SD. Nurr1 expression was verified by western blotting. [file Image1.TIFF]
